# Concurrent COVID-19 and Tuberculosis in an Immigrant Worker Presenting with Hemoptysis

**DOI:** 10.4269/ajtmh.21-0477

**Published:** 2021-07-16

**Authors:** Toshinori Sahara, Kazuhisa Yokota

**Affiliations:** Department of Infectious Diseases, Ebara Hospital, Tokyo Metropolitan Health and Hospital Corporation, Tokyo, Japan

**Figure 1.**
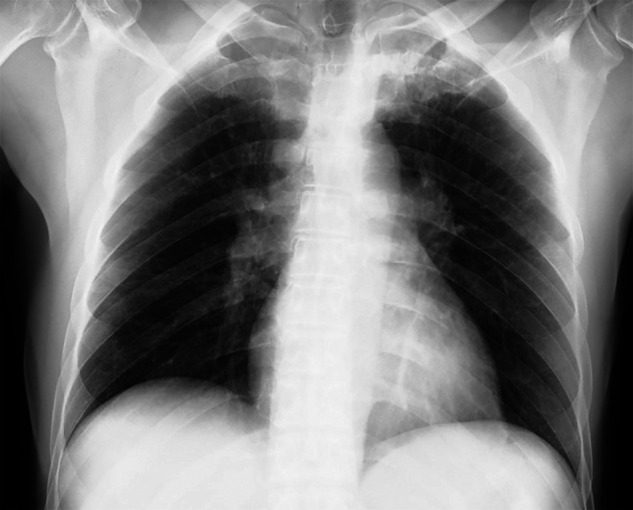
Chest X-ray on admission.

**Figure 2.**
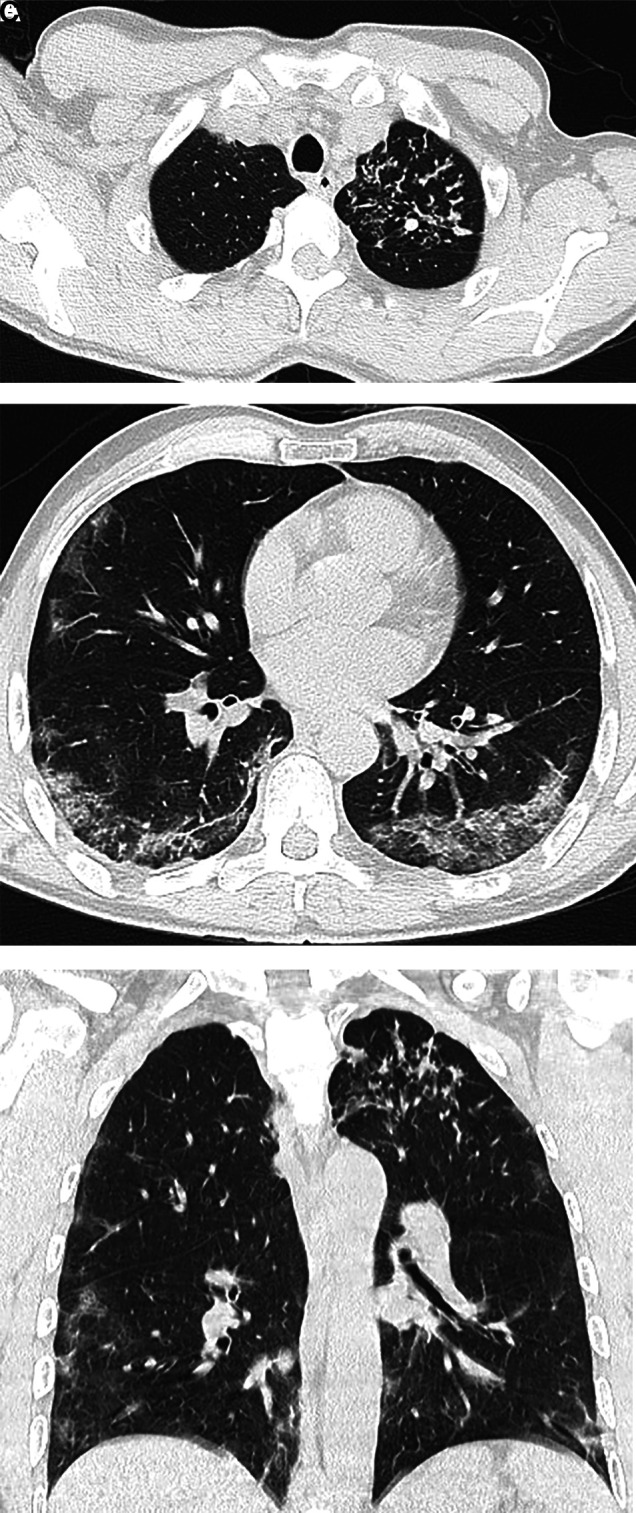
(**A**) Tree-in-bud signs in the left upper lobe. (**B**) Bilateral ground-glass opacities in a peripheral distribution. (**C**) Coronal computed tomographic scan shows both tree-in-bud pattern and peripheral ground-glass opacities.

A 59-year-old man was referred to our hospital by a regional public health center for the treatment of coronavirus disease 2019 (COVID-19), which was diagnosed by the nasopharyngeal swab polymerase chain reaction test. The patient, who is immigrant worker from Nepal at an Indian restaurant in Japan, had acute-onset fever, productive cough with hemoptysis, and taste and smell disorders for 8 days before admission. He had no past medical history including tuberculosis (TB), and no subacute or chronic symptoms to suspect TB. None of his family members had TB. When he was admitted, he had a body temperature of 37.4°C, a respiratory rate of 14 breaths/min, an oxygen saturation level of 100%, and his chest radiograph showed clear lungs (Figure 1). He used acetaminophen and dextromethorphan hydrobromide hydrate to control his fever and cough, but his body temperature proceeded to exceed 38°C for 5 days after hospitalization, his productive cough with hemoptysis worsened, and although his oxygen saturation level did not decline, he gradually lost his appetite. We therefore elected to examine his sputum at day 5 after admission. Initial acid-fast stain smears were negative for 3 consecutive days, but the patient finally tested positive for TB by our in-house laboratory polymerase chain reaction analysis of his sputum. Chest computed tomographic scans performed on day 8 after admission revealed bilateral ground-glass opacities, which are typical for COVID-19 patients, and a tree-in-bud pattern in the left upper lobe showing active TB (Figure 2). There were no computed tomographic findings to suspect hilar and mediastinal lymphadenopathy.

After diagnosis of TB, the patient was treated using four standard drugs (rifampicin, isoniazid, ethambutol, and pyrazinamide) from day 9 after admission. His fever decreased the following day, his productive cough improved gradually, and his appetite returned. The sputum culture subsequently tested positive for *Mycobacterium tuberculosis*, which was sensitive to every anti-TB drug.

The prevalence of hemoptysis in patients with COVID-19 and TB has been reported as 2%^1^ and as approximately 8%.^2^^,^^3^ Elise et al.^4^ reported a COVID-19 case with acute-onset hemoptysis, and another report indicated that the duration of hemoptysis in patients with COVID-19 may be less than 10 days.^5^

This case exemplifies the importance of being aware of possible concurrent infections in patients with COVID-19, and that correct assessment of patients at appropriate times is essential to identify co-infections. The epidemiological background of TB, clinical pattern of symptom onset, and period of hemoptysis are key to recognizing concurrent infection of TB among patients with COVID-19 presenting hemoptysis.
